# Stability-Controlled Continual Federated Learning for Energy-Harvesting AIoT Systems

**DOI:** 10.3390/s26113325

**Published:** 2026-05-23

**Authors:** Junsoo Park, Ikjune Yoon, Dong Kun Noh

**Affiliations:** 1 School of Biomedical Systems, Soongsil University, Seoul 06978, Republic of Korea; freefree9758@naver.com; 2 Division of AI Computer Science & Engineering, Kyonggi University, Suwon 16227, Republic of Korea; ijyoon@kyonggi.ac.kr; 3 School of AI Convergence, Soongsil University, Seoul 06978, Republic of Korea

**Keywords:** energy-harvesting AIoT, federated learning, continual learning, energy-aware control, Lyapunov stability

## Abstract

Energy-harvesting (EH) AIoT systems enable long-term autonomous operation but suffer from time-varying energy availability, which makes stable learning difficult. In such environments, federated learning (FL) is prone to energy depletion (blackout), while continual learning is required to handle evolving data distributions, leading to a trade-off between energy stability and catastrophic forgetting. In this paper, we propose a stability-controlled continual federated learning framework that jointly regulates local training intensity and rehearsal usage based on the residual energy state. The proposed method is derived from a Lyapunov drift-plus-penalty formulation and implemented as a lightweight mode-based control policy. Simulation results using real solar energy traces show that the proposed method significantly reduces blackout while improving accuracy and mitigating forgetting compared to existing approaches. These results demonstrate the effectiveness of energy-aware joint control for stable continual federated learning in EH-AIoT systems.

## 1. Introduction

Energy-harvesting (EH) AIoT systems have attracted significant attention as a key enabler of long-term autonomous edge intelligence. By harvesting energy from ambient sources such as solar, vibration, and thermal gradients, these systems can operate without frequent battery replacement, making them suitable for deployment in hard-to-access environments, including environmental monitoring, smart agriculture, and industrial safety applications. Energy harvesting and system-level optimization have been extensively studied in prior works, particularly in renewable-energy harvesting, solar-powered, and wireless-powered IoT/sensor systems [1,2,3,4,5,6], highlighting the feasibility and potential of sustainable IoT systems. Among various energy sources, solar energy is the most widely adopted due to its relatively high power density and availability. However, despite its advantages, solar energy exhibits strong temporal variability depending on day–night cycles, weather conditions, seasonal changes, and installation factors. As a result, the amount of available energy fluctuates significantly over time, leading to continuously varying energy budgets at edge devices.

A representative application scenario is a solar-powered AIoT monitoring system deployed in remote fields, industrial facilities, or outdoor infrastructure. In such systems, distributed sensor nodes continuously collect local observations and perform on-device learning or inference under limited and time-varying harvested energy. The sensed data may also evolve over time due to weather changes, seasonal patterns, crop growth stages, equipment aging, or newly emerging events. Therefore, each device must handle two coupled dynamics: fluctuating energy availability and non-stationary local data distributions.

This energy variability poses a fundamental challenge for learning-enabled AIoT systems. If computation, communication, and learning are performed at a constant rate without considering the energy state, the device may experience repeated energy depletion, resulting in blackout. Blackout is not merely a temporary shutdown; it can lead to data loss, interrupted sensing, failed model updates, and degraded system reliability. In learning-based systems, repeated blackout events can significantly hinder model convergence and degrade long-term performance. Therefore, maintaining energy stability while performing learning tasks is a critical requirement in EH-AIoT systems.

Federated learning (FL) has emerged as an effective paradigm for distributed learning in AIoT environments [7,8,9]. In FL, each device performs local training using its private dataset and periodically transmits model updates to a central server for aggregation. This approach preserves data privacy and reduces communication overhead compared to centralized learning. However, FL inherently incurs energy costs from both local computation and communication. In EH environments, where devices have heterogeneous and time-varying energy availability, some nodes may be unable to participate in training due to insufficient energy. This leads to partial participation, biased updates, and degraded generalization performance of the global model.

In addition to energy constraints, real-world AIoT systems operate in dynamic environments where data distributions evolve over time. Sensor observations may change due to environmental conditions, usage patterns, or the emergence of new events and classes. To handle such non-stationary data, continual learning is required, where the model incrementally learns new information while retaining previously acquired knowledge. However, continual learning suffers from the well-known problem of catastrophic forgetting, in which previously learned knowledge is gradually overwritten during sequential training [10,11,12]. Rehearsal-based methods, which reuse a subset of past data during training, are widely adopted to mitigate forgetting. Nevertheless, rehearsal introduces additional computational and memory overhead, thereby increasing energy consumption.

Consequently, EH-AIoT systems with continual federated learning face a fundamental trade-off between energy stability and learning performance. Increasing the local training intensity or rehearsal usage improves model accuracy and reduces forgetting but leads to higher energy consumption and increased risk of blackout. Conversely, reducing computation to preserve energy may stabilize the system but degrades learning performance and accelerates knowledge loss. This trade-off is not adequately addressed by existing approaches, which typically consider either energy efficiency or learning performance in isolation.

This problem cannot be directly solved by simply applying existing energy-aware federated learning or federated continual learning methods. Energy-aware FL approaches mainly focus on reducing communication cost, selecting energy-sufficient clients, or adapting training effort under resource constraints, but they generally do not explicitly consider catastrophic forgetting or the additional energy cost caused by rehearsal. In contrast, federated continual learning methods are designed to handle non-stationary data and preserve previously learned knowledge, but they typically assume stable power availability and do not directly address energy neutrality or blackout risk in energy-harvesting devices. This gap motivates a unified framework that jointly regulates learning intensity and rehearsal usage according to the residual energy state.

To address this challenge, this paper formulates the interaction between energy dynamics and continual federated learning as a unified control problem. Instead of treating energy management and learning optimization separately, we explicitly couple them through controllable variables, namely the local training intensity and rehearsal ratio. Based on this formulation, we propose a stability-controlled continual federated learning framework that dynamically adjusts these variables according to the residual energy state of each device. The proposed approach is grounded in a Lyapunov drift-plus-penalty framework, which provides a principled method for balancing energy stability and learning performance. This formulation enables the system to reduce energy consumption when the residual energy is low and to increase learning effort when sufficient energy is available.

To ensure practical applicability in resource-constrained devices, we further design a lightweight mode-based control policy that approximates the optimal control behavior derived from the Lyapunov framework. The policy operates with minimal computational overhead and can be implemented efficiently in real-world systems. By integrating energy-aware control with continual federated learning, the proposed framework achieves stable long-term operation while maintaining high learning performance.

Unlike prior studies that address energy-aware control and continual learning separately, the proposed framework provides a unified stability–theoretic formulation that explicitly couples energy dynamics with continual learning behavior. In particular, it jointly regulates training intensity and rehearsal usage under a Lyapunov-based control principle, enabling simultaneous optimization of energy stability and long-term learning performance.

The main contributions of this paper are summarized as follows:

- We formulate continual federated learning in EH-AIoT as a unified control problem that captures the trade-off between energy stability and forgetting.
- We propose a stability-controlled policy that jointly adapts local training intensity and rehearsal ratio based on residual energy.
- We derive the control policy from a Lyapunov drift-plus-penalty framework, providing theoretical grounding for stability-aware learning.
- We design a lightweight mode-based implementation suitable for resource- constrained devices.
- We demonstrate improved energy stability and learning performance through solar trace-based simulations.

## 2. Related Work

In this section, we review existing approaches related to federated learning in energy-constrained environments and continual learning techniques, with a focus on their limitations in energy-harvesting (EH) AIoT systems. Prior studies can be broadly categorized into four groups: (i) fixed training schemes, (ii) energy-aware participation control, (iii) continual learning techniques, and (iv) model-efficiency approaches.

### 2.1. Fixed Training Schemes

A common approach in federated learning is to use a fixed local training configuration, where each client performs a predetermined number of local steps or epochs in every round [7,8]. This approach is simple to implement and widely used due to its predictable behavior and ease of analysis. However, in EH environments with time-varying energy availability, fixed training schemes fail to adapt to changing energy conditions. When sufficient energy is available, the system may operate efficiently, but during low-energy periods, such as nighttime or cloudy conditions in solar-powered systems, the same training intensity can rapidly deplete the available energy.

As a result, repeated blackout events may occur, leading to missed updates, communication failures, and degraded convergence of the global model. Moreover, since energy depletion patterns are often periodic in EH systems, such failures can accumulate over time, significantly affecting long-term system stability and learning performance. Therefore, fixed training schemes are not suitable for EH-AIoT environments where adaptive energy-aware operation is required.

### 2.2. Energy-Aware Participation Control

To address energy limitations, several studies have proposed energy-aware participation control mechanisms. A widely used strategy is threshold-based participation, where a client participates in training only if its residual energy exceeds a predefined threshold [13,14]. This approach effectively prevents severe energy depletion by disabling training during low-energy conditions.

Beyond simple threshold-based participation, recent EH-FL studies explicitly model the interaction between harvested energy, device participation, and wireless transmission. For example, MDP-based EH-FL formulations jointly optimize device scheduling and transmit power under stochastic harvested energy and channel conditions [15]. Such methods are effective for improving convergence behavior and communication reliability in energy-constrained FL systems because they account for battery state, packet drops, and wireless channel variation. However, their control objective is mainly associated with participation and communication decisions, and they do not explicitly model continual learning behavior, rehearsal-induced computation cost, or catastrophic forgetting.

Despite its simplicity, threshold-based methods suffer from several limitations. First, they only control whether a client participates in training, without adjusting the intensity of learning. This binary decision mechanism lacks flexibility and may result in under-utilization of available energy. Second, these approaches do not consider the additional cost associated with rehearsal or continual learning, making them insufficient in dynamic learning scenarios. Third, frequent switching between participation and non-participation near the threshold can cause unstable training behavior. Finally, the threshold is typically determined empirically, without a theoretical basis for ensuring long-term stability.

Consequently, while threshold-based control improves energy stability to some extent, it often sacrifices learning continuity and performance.

### 2.3. Continual Learning in Dynamic Environments

Continual learning has been extensively studied to address non-stationary data distributions, where models must adapt to new tasks while preserving previously learned knowledge [10,11,12]. Among various approaches, rehearsal-based methods are widely adopted due to their effectiveness in mitigating catastrophic forgetting [16,17,18,19]. These methods store a subset of past data and replay it during training, thereby maintaining performance on previous tasks. In addition to these core approaches, various extensions of continual learning, including improved replay strategies and federated continual learning, have also been explored in recent studies [20,21,22].

Federated continual learning (FCL) extends continual learning to distributed clients whose local data distributions evolve over time. Recent FCL methods address temporal non-stationarity and client heterogeneity using mechanisms such as knowledge fusion, memory-based rehearsal, prototype or representation sharing, and uncertainty-aware memory management [21,22,23]. These methods are closely related to our work because they aim to preserve knowledge under non-IID and temporally changing client data. However, their main focus is learning stability under data drift, and they generally do not explicitly model residual energy dynamics, harvested-energy uncertainty, energy-neutral operation, or blackout risk at the client side. In addition, rehearsal is usually treated as a learning mechanism, rather than as an energy-consuming control variable that must be adapted according to the device energy state.

More importantly for EH-AIoT systems, applying continual learning mechanisms without energy-aware control remains problematic. In EH-AIoT systems, rehearsal introduces additional computational and memory overhead, directly increasing energy consumption. As a result, applying continual learning methods without energy awareness can lead to increased blackout frequency, especially during low-energy periods. This creates a conflict between maintaining model performance and preserving system stability.

Therefore, conventional continual learning approaches are not directly applicable to EH environments, where resource constraints must be explicitly considered.

### 2.4. Model Efficiency and Lightweight Approaches

To reduce computational and communication costs, resource-aware federated learning techniques such as adaptive training, edge-assisted learning, and lightweight model operation have been widely explored [13,14]. These approaches aim to reduce learning and communication overhead, thereby lowering energy consumption.

While such techniques are effective in reducing per-round cost, they do not directly address the trade-off between energy stability and knowledge retention. In particular, reducing model complexity does not inherently prevent catastrophic forgetting, nor does it provide a mechanism to adapt learning behavior based on energy availability. Furthermore, combining lightweight models with rehearsal-based continual learning may still incur additional energy costs, depending on the training configuration.

Thus, model-efficiency techniques alone are insufficient to resolve the fundamental trade-off in EH-AIoT systems.

### 2.5. Summary and Motivation

Table 1 summarizes the technical relationship between the proposed framework and representative related research directions. The comparison highlights that existing methods usually address energy-aware FL, EH-FL scheduling, or federated continual learning separately, whereas the proposed framework jointly considers energy harvesting, continual learning, rehearsal control, and stability-aware energy management.

In summary, existing approaches address important but separate aspects of the target problem. Energy-aware FL and EH-FL methods improve client participation, communication efficiency, and scheduling under energy constraints, but they do not explicitly control rehearsal usage or catastrophic forgetting. In contrast, federated continual learning methods improve knowledge retention under non-stationary data distributions, but they generally do not account for harvested-energy dynamics, blackout risk, or energy-neutral operation. Model-efficiency techniques can reduce per-round computational or communication cost, but they do not determine when and how much continual learning effort should be performed under time-varying energy availability. Therefore, none of these approaches directly addresses the coupled problem of energy stability and continual learning in EH-AIoT systems.

These limitations highlight the need for a unified control framework that jointly considers energy dynamics and continual learning objectives. In particular, a mechanism that adaptively regulates both training intensity and rehearsal usage based on the residual energy state is required to balance energy stability and long-term learning performance. This motivates the stability-controlled continual federated learning approach proposed in this paper.

## 3. Proposed Method

**Figure 1:**
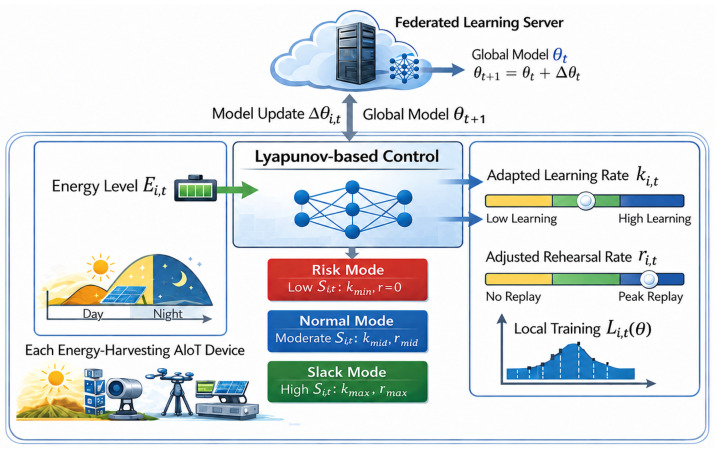
Overview of the proposed stability-controlled continual federated learning framework for energy-harvesting AIoT systems.

Figure 1 presents the overall architecture of the proposed framework. The key idea of this work is to treat *energy stability* and *continual learning performance* as a coupled control problem rather than two independent design goals. In an energy-harvesting AIoT environment, each client experiences a time-varying energy budget determined by the harvested energy and the residual battery level. At the same time, the client must continuously learn from newly arriving data while retaining previously acquired knowledge. These two requirements are inherently coupled because stronger learning typically consumes more energy, whereas excessive energy saving often degrades learning quality and increases forgetting.

In Figure 1, the learning-rate and rehearsal-rate bars on the right side indicate three discrete control levels corresponding to the Risk, Normal, and Slack modes, rather than a continuous or finer-grained adjustment process.

To address this issue, the proposed framework introduces an energy-aware stability controller at each client. Based on the current residual energy and the expected short-term harvested energy, the controller determines two decision variables: the local training intensity and the rehearsal ratio. The local training intensity controls how much learning is performed in the current round, whereas the rehearsal ratio controls how much past data are reused to mitigate forgetting. These decisions directly affect both the energy consumption and the learning outcome. Therefore, by jointly controlling these two variables, the proposed method aims to maintain stable long-term operation while preserving continual learning performance.

The proposed method consists of four major components: (i) system and energy modeling, (ii) continual learning-aware local objective formulation, (iii) Lyapunov-based stability control [24], and (iv) a practical three-level control policy for real deployment. The overall flow is as follows. First, each client observes its current energy state and predicts near-future harvested energy. Second, the controller determines an appropriate operating mode. Third, according to the selected mode, the client sets its training intensity and rehearsal ratio. Finally, the client first checks whether the selected local training and transmission process is energy-feasible; only when this condition is satisfied does the client perform local learning and upload the resulting model update. This process is repeated at every FL round.

### 3.1. System Model

We consider a federated learning system composed of one central server and *N* energy-harvesting clients. At communication round *t*, the server broadcasts the global model $\theta_{t}$ to a subset of clients. Each participating client *i* trains the received model using its local data and returns a local update $\Delta \theta_{i,t}$ to the server. The server then aggregates the received updates, for example through FedAvg [7], to produce the next global model $\theta_{t+1}$.

Unlike conventional FL settings, each client in our system is powered by harvested energy and therefore cannot be assumed to have a stable power supply. This makes the client state highly dynamic. Specifically, the client must decide whether it can afford aggressive local learning, limited learning, or only minimal maintenance-level operation in a given round.

In addition, the local data distribution is assumed to evolve over time. This reflects realistic AIoT scenarios in which the sensed environment changes due to season, time, usage pattern, or the appearance of new event classes. Hence, the client does not merely solve a static classification problem, but rather a continual learning problem in which newly observed patterns must be incorporated without destroying previously acquired knowledge.

Each client maintains the following local resources:

- A residual energy state $E_{i,t}$;
- A current local dataset $D_{i,t}^{cur}$;
- A replay buffer $D_{i,t}^{buf}$ that stores a subset of past samples;
- A local controller that selects the training configuration for the current round.

The role of the controller is central in our framework. Instead of always using a fixed learning configuration, the controller adaptively adjusts the amount of learning according to the energy condition. This is the main mechanism that connects energy-harvesting dynamics with continual federated learning.

### 3.2. Energy Dynamics and Local Learning Objective

The residual energy of client *i* evolves according to the following battery dynamics:

(1) $$E_{i,t+1}=\min\left(C_{\max},E_{i,t}+H_{i,t}-C_{i,t}\right),$$

where $E_{i,t}$ denotes the residual energy at round *t*, $H_{i,t}$ is the harvested energy during that round, $C_{i,t}$ is the consumed energy, and $C_{\max}$ is the battery capacity. A blackout occurs when the available energy becomes insufficient to support the required local operation. In practice, blackout means that local training or update transmission fails, which may cause missing participation, distorted aggregation, and unstable model evolution.

Equation (1) shows why energy-aware control is necessary. If the learning cost $C_{i,t}$ is repeatedly larger than the amount of replenished energy, the residual energy will continuously decrease. Therefore, a stable method must not only aim for good accuracy, but must also keep the long-term energy trajectory within a safe range.

To model continual learning, we define the local objective as

(2) $$L_{i,t}\left(\theta \right)=L_{i,t}^{cur}\left(\theta \right)+\lambda r_{i,t}L_{i,t}^{buf}\left(\theta \right),$$

where $L_{i,t}^{cur}\left(\theta \right)$ is the loss on the current data, $L_{i,t}^{buf}\left(\theta \right)$ is the loss on replayed buffer samples, λ is the buffer-loss weighting factor, and $r_{i,t}\in \left[0,r_{\max}\right]$ is the rehearsal ratio. The rehearsal ratio represents the relative proportion of replayed buffer samples used during local training to mitigate catastrophic forgetting. In the proposed mode-based implementation, $r_{i,t}$ is selected from $\left\{0,r_{\mathrm{mid}},r_{\max}\right\}$ according to the operating mode.

This formulation is important because it explicitly captures the trade-off between adaptation and retention. The first term allows the model to adapt to newly observed data, while the second term preserves past knowledge through replay. A larger $r_{i,t}$ generally improves resistance to catastrophic forgetting, but it also requires additional forward and backward computation. Therefore, the rehearsal ratio cannot be chosen independently of the energy condition.

The local learning cost is modeled as

(3) $$C_{i,t}=C_{base}+C_{i,t}^{train}\left(k_{i,t},r_{i,t}\right)+C_{i,t}^{comm}\left(s_{\Delta }\right),$$

where $C_{base}$ is the base energy consumption of sensing and system maintenance, $C_{i,t}^{train}$ is the training cost, and $C_{i,t}^{comm}$ is the communication cost for update transmission.

We approximate the training energy as

(4) $$C_{i,t}^{train}\left(k_{i,t},r_{i,t}\right)\approx ak_{i,t}\left(1+\rho r_{i,t}\right),$$

where $k_{i,t}$ denotes the local training intensity, which represents the number or relative amount of local training steps performed by client *i* at round *t*, *a* is the per-step energy coefficient, and ρ reflects the additional cost caused by replay mixing. In the proposed mode-based implementation, $k_{i,t}$ is selected from the discrete set $\left\{k_{\min},k_{\mathrm{mid}},k_{\max}\right\}$. The communication energy is approximated as

(5) $$C_{i,t}^{comm}\left(s_{\Delta }\right)\approx bs_{\Delta },$$

where $s_{\Delta }$ is the size of the transmitted update and *b* is the energy cost per data unit.

The energy-cost models in Equations (3)–(5) are adopted as first-order linear approximations of the dominant energy-consumption factors in local learning and update transmission. This modeling choice provides a lightweight and interpretable formulation for deriving the proposed control policy on resource-constrained EH-AIoT devices, while avoiding device-specific calibration complexity. Although real hardware platforms may exhibit nonlinear energy behavior depending on processor state, memory access, wireless channel condition, and hardware implementation, such nonlinear models can be incorporated into the same framework when detailed profiling data are available.

These equations clarify the role of the two control variables. Increasing $k_{i,t}$ means performing more local learning, which may improve convergence but increases energy usage. Increasing $r_{i,t}$ helps prevent forgetting but also increases energy consumption. Therefore, the control problem of this paper can be summarized as follows: *how should a client choose $k_{i,t}$ and $r_{i,t}$ at every round so that long-term energy stability is preserved without sacrificing continual learning performance?*

### 3.3. Why Stability Control Is Needed

The energy and learning-cost models above show that the training intensity $k_{i,t}$ and rehearsal ratio $r_{i,t}$ directly affect both energy consumption and continual learning performance. Therefore, a client should not simply decide whether to participate in training, but should determine how much local learning and rehearsal can be safely performed under the current energy condition.

A fixed training configuration is unsuitable in EH-AIoT systems because the available energy varies over time. A configuration that is feasible during high-harvesting periods may cause energy depletion during low-harvesting periods. Similarly, binary participation control is too coarse because it only switches between full participation and no participation, without adjusting the learning configuration itself.

For this reason, the proposed method adopts a stability-control perspective. The goal is to guide the learning configuration according to the residual energy state so that the system avoids energy collapse while still maintaining sufficient continual learning capability. This motivates the Lyapunov-based formulation introduced in the next subsection.

### 3.4. Lyapunov-Based Stability Formulation

To formally capture energy stability, we define the following Lyapunov function:

(6) $$V\left(E_{i,t}\right)=\frac{1}{2}{\left(E_{i,t}-E^{*}\right)}^{2},$$

where $E^{*}$ denotes the target energy level. This function measures how far the current residual energy deviates from the desired operating point. If $E_{i,t}$ remains close to $E^{*}$, the system is considered stable. If the deviation becomes large, the system moves toward an undesirable operating region.

The one-step Lyapunov drift is defined as

(7) $$\Delta V=V\left(E_{i,t+1}\right)-V\left(E_{i,t}\right).$$

By substituting the energy dynamics from (1), we obtain

(8) $$\Delta V=\left(E_{i,t}-E^{*}\right)\left(H_{i,t}-C_{i,t}\right)+\frac{1}{2}{\left(H_{i,t}-C_{i,t}\right)}^{2}.$$

This equation provides the core intuition of the proposed method. Consider the case where the client has low residual energy, i.e., $E_{i,t}<E^{*}$. Then, the term $\left(E_{i,t}-E^{*}\right)$ becomes negative. In this case, increasing the energy consumption $C_{i,t}$ tends to increase the drift, which pushes the system away from the desired stable operating point. Therefore, when the client is energy-poor, the control policy should reduce the learning cost. In contrast, when $E_{i,t}>E^{*}$, the client has enough energy margin, and stronger learning can be allowed.

To jointly account for stability and learning performance, we adopt the drift-plus-penalty principle:

(9) $$\min_{k_{i,t},r_{i,t}}\;\Delta V+\alpha L_{i,t}\left(\theta \right),$$

where α is a balancing parameter. Here, α≥0 controls the relative importance of the learning-loss term in the drift-plus-penalty objective. A larger α places more emphasis on reducing the learning loss, whereas a smaller α gives relatively higher priority to energy stability. Since the Lyapunov drift and learning-loss terms may have different numerical scales depending on the system configuration and loss normalization, α does not have a fixed universal upper bound and can be selected empirically according to the desired stability–performance trade-off. The first term encourages energy stability, while the second term encourages learning quality. This formulation is useful because it directly connects the control variables with the system objectives. The method does not optimize only accuracy or only battery survival; it optimizes both within a single framework.

To obtain a tractable per-round control objective, we separate the terms in the drift expression according to whether they depend on the current control decision. At round *t*, the residual energy $E_{i,t}$, target energy level $E^{*}$, and harvested energy $H_{i,t}$ are treated as observed or exogenous quantities. Therefore, terms that depend only on $E_{i,t}$, $E^{*}$, and $H_{i,t}$ do not affect the minimization over the decision variables $k_{i,t}$ and $r_{i,t}$ and can be treated as constants in the per-round optimization. In contrast, the energy consumption $C_{i,t}$ depends on the selected local training intensity and rehearsal ratio through Equations (3)–(5), and therefore remains decision-dependent.

Accordingly, by omitting the decision-independent terms and retaining the learning-performance penalty, the drift-plus-penalty objective can be interpreted in the following simplified control-oriented form:


(10) $$\min_{k_{i,t},r_{i,t}}\;\alpha L_{i,t}\left(\theta \right)+\left(E_{i,t}-E^{*}\right)C_{i,t}.$$

The learning-performance term $\alpha L_{i,t}\left(\theta \right)$ is retained because the selected training intensity and rehearsal ratio directly affect the local learning outcome. The harvested-energy-related terms are omitted from the simplified decision objective because harvested energy is not controlled by the client in the current round. The resulting objective is minimized subject to the feasible control set used by the proposed mode-based policy, namely $k_{i,t}\in \left\{k_{\min},k_{\mathrm{mid}},k_{\max}\right\}$ and $r_{i,t}\in \left\{0,r_{\mathrm{mid}},r_{\max}\right\}$, together with the energy-feasibility condition for the selected local training and transmission operation.

This interpretation is particularly intuitive. When the residual energy is low, the coefficient on $C_{i,t}$ discourages aggressive learning. When the residual energy is high, the controller can place relatively more emphasis on reducing the learning loss. Thus, the control policy naturally adapts the training configuration to the current energy state.

### 3.5. Justification for Three-Level Control

Although the formulation in (9) is conceptually clean, solving it exactly at every round is not practical for resource-constrained edge devices. The client would need to evaluate multiple candidate configurations, estimate their future effect, and solve an optimization problem online. This is undesirable in the very setting where computational efficiency matters.

For this reason, we design a *three-level control policy* that approximates the behavior implied by the Lyapunov formulation while remaining simple enough for practical deployment.

First, we define the energy slack as

(11) $$S_{i,t}=E_{i,t}+{\hat{H}}_{i,t}-E_{safe},$$

where ${\hat{H}}_{i,t}$ is the predicted near-future harvested energy and $E_{safe}$ is a safety threshold. The slack value represents the short-term energy margin available for learning. If this value is negative or very small, the client should protect its battery. If it is sufficiently large, the client may use the opportunity to learn more aggressively.

In the proposed framework, the near-future harvested energy ${\hat{H}}_{i,t}$ is estimated using a lightweight moving-average method based on the recent harvested-energy history. Specifically, the client computes the short-term energy estimate from the harvested-energy observations during the previous several rounds:

(12) $${\hat{H}}_{i,t}=\frac{1}{L}\sum_{\ell =1}^{L}H_{i,t-\ell },$$

where *L* denotes the observation window size. This estimation is intentionally designed as a lightweight short-term predictor for energy-aware mode selection rather than a complex long-term solar forecasting model. Since the proposed controller operates on resource-constrained EH-AIoT devices, minimizing prediction overhead is an important design consideration.

The proposed framework does not assume perfect prediction of future harvested energy. The estimated value ${\hat{H}}_{i,t}$ is used only to compute the short-term energy slack for mode selection. Since the controller maps the slack value to three discrete operating modes rather than continuously adjusting the control variables, small prediction errors do not necessarily cause abrupt changes in the learning configuration. In this sense, the mode-based policy provides a lightweight and robust mechanism for handling moderate uncertainty in harvested-energy estimation. Large prediction errors may still affect mode selection, and incorporating more advanced forecasting models remains a possible extension.

A key design question is why we use *three* levels rather than a binary rule or a fully continuous controller. The binary design is too coarse. It can distinguish only between “train” and “do not train,” which is insufficient in our setting because many rounds fall into an intermediate region. In those rounds, completely stopping learning wastes useful energy, while fully aggressive training may still be unsafe. Therefore, an intermediate operating mode is necessary.

On the other hand, a fully continuous controller is more difficult to implement robustly on constrained devices. It requires finer parameter calibration, more computation, and potentially causes unstable oscillatory behavior in the presence of noisy energy prediction. Since the harvested energy is stochastic and imperfectly predictable, a simpler and more robust policy is preferable.

Therefore, the proposed three-level policy provides a practical compromise between expressiveness and simplicity:

- **Risk mode** is selected when $S_{i,t}\leq 0$, indicating that the predicted short-term energy margin is insufficient. In this mode, the client uses the minimum training intensity and disables rehearsal to protect the battery and reduce blackout risk.
- **Normal mode** is selected when $0<S_{i,t}<S_{\mathrm{high}}$, indicating that the client has a usable but limited energy margin. In this mode, the client performs moderate training and rehearsal to maintain learning continuity without aggressive energy consumption.
- **Slack mode** is selected when $S_{i,t}\geq S_{\mathrm{high}}$, indicating that sufficient energy margin is available. In this mode, the client increases both training intensity and rehearsal usage to improve adaptation and knowledge retention.

Formally, the operating mode is selected as follows:

(13) $${\mathrm{Mode}}_{i,t}=\left\{\begin{array}{cc} \mathrm{Risk}, & S_{i,t}\leq 0,\\ \mathrm{Normal}, & 0<S_{i,t}<S_{high},\\ \mathrm{Slack}, & S_{i,t}\geq S_{high}, \end{array}\right.$$

where $S_{\mathrm{high}}$ is the threshold above which the client is considered to have sufficient additional energy margin for aggressive local training and rehearsal. The two thresholds, 0 and $S_{\mathrm{high}}$, divide the energy-slack space into three operational regions: an unsafe or energy-deficient region, an intermediate region with limited but usable energy, and an energy-abundant region.

The local training intensity is then chosen by

(14) $$k_{i,t}=\left\{\begin{array}{cc} k_{\min}, & \mathrm{Risk}\;\mathrm{mode},\\ k_{\mathrm{mid}}, & \mathrm{Normal}\;\mathrm{mode},\\ k_{\max}, & \mathrm{Slack}\;\mathrm{mode}, \end{array}\right.$$

and the rehearsal ratio is selected by

(15) $$r_{i,t}=\left\{\begin{array}{cc} 0, & \mathrm{Risk}\;\mathrm{mode},\\ r_{\mathrm{mid}}, & \mathrm{Normal}\;\mathrm{mode},\\ r_{\max}, & \mathrm{Slack}\;\mathrm{mode}. \end{array}\right.$$

This structure has several advantages. First, it is computationally lightweight because it requires only slack computation and simple comparisons. Second, it is interpretable because each mode has a clear physical meaning. Third, it is robust because small fluctuations in energy prediction do not immediately lead to large control changes. Finally, it remains expressive enough to reflect the asymmetric needs of EH continual learning: strong protection at low energy, balanced learning in moderate conditions, and aggressive knowledge consolidation when energy is abundant.

### 3.6. Algorithm Description

The complete client-side procedure of the proposed method is summarized in Algorithm 1. The algorithm begins by measuring the current residual energy and estimating the short-term harvested energy. Based on these values, the client computes its energy slack and determines the operating mode. The selected mode specifies both the local training intensity and the rehearsal ratio.

Before constructing local mini-batches and executing local training, the client estimates the expected energy cost of the selected configuration, including both training and transmission costs. If the selected operation is energy-feasible, the client performs local training and transmits the resulting model update; otherwise, it skips the local update in the current round to avoid wasting energy on an update that cannot be delivered.

The harvested-energy estimation process uses a moving-average predictor over recent harvested-energy observations, which introduces negligible computational overhead compared to the local neural network training process. This lightweight design allows the proposed framework to operate efficiently on practical EH-AIoT edge devices while still providing sufficient short-term energy awareness for adaptive mode selection.

### 3.7. Theoretical Interpretation

The proposed control policy provides two important theoretical properties.

First, it promotes long-term energy boundedness. In the Risk mode, the policy selects the minimum-cost configuration by setting $k_{i,t}=k_{\min}$ and $r_{i,t}=0$. Let the corresponding lower-bounded energy cost be

(16) $$C_{\min}=C_{base}+ak_{\min}+bs_{\Delta }.$$

If the long-term average harvested energy satisfies

(17) $$E\left[H_{t}\right]>E\left[C_{\min}\right],$$

then the client can avoid persistent energy collapse because the controller reduces the learning load whenever the system approaches a low-energy region. In this sense, the policy enforces an energy-neutral operating tendency.

**Algorithm 1:** Stability-Controlled Continual Federated Learning at Client *i*.

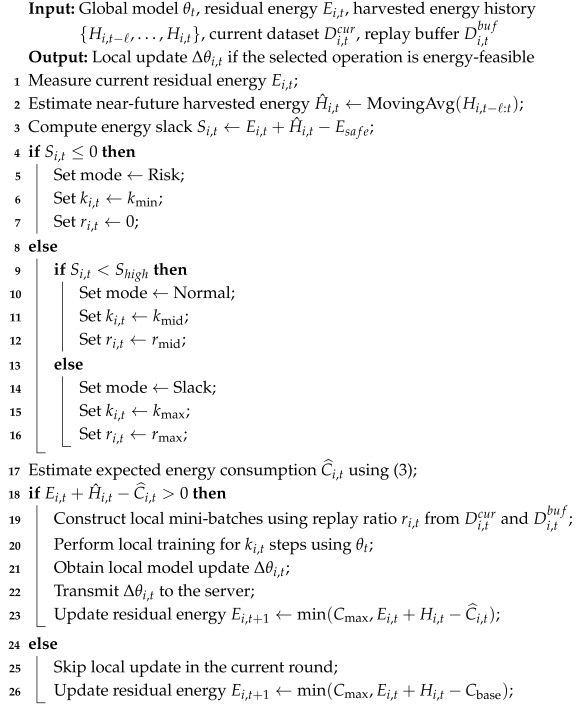



Second, the policy improves continual learning quality in an energy-aware manner. In high-energy rounds, the Slack mode increases both $k_{i,t}$ and $r_{i,t}$, which allows stronger adaptation and better replay of past knowledge. In low-energy rounds, replay is suppressed to avoid wasting scarce energy on expensive memory-based training. This means that rehearsal is applied when it is most affordable rather than uniformly across all rounds. As a result, the proposed method can mitigate catastrophic forgetting without sacrificing stability.

From a stability perspective, the proposed control policy ensures that the residual energy remains bounded over time under mild conditions on the average harvested energy. This property follows from the Lyapunov-based formulation, which penalizes excessive energy consumption when the system approaches low-energy states, thereby preventing long-term energy collapse.

In summary, the proposed method is not a heuristic collection of rules. It is a practically implementable approximation of a Lyapunov-guided stability control principle, specifically tailored to the characteristics of continual federated learning in energy-harvesting AIoT systems.

## 4. Experimental Evaluation

This section presents a comprehensive simulation-based evaluation of the proposed stability-controlled continual federated learning framework. The objective is to verify whether the proposed method can simultaneously achieve (i) long-term energy stability, (ii) high learning performance, and (iii) effective mitigation of catastrophic forgetting under realistic energy-harvesting conditions.

The proposed framework was evaluated using solar trace-based simulations rather than deployment on a physical hardware prototype. This simulation-based approach allows controlled analysis of the interaction between energy dynamics, local learning behavior, and continual learning performance under time-varying harvested-energy conditions.

To broaden the simulation evaluation, we consider two energy-harvesting scenarios: a baseline scenario using the original NREL NSRDB solar trace and an additional stochastic scenario in which random perturbations are applied to the solar trace to emulate practical environmental fluctuations.

### 4.1. Experimental Setup

#### 4.1.1. Solar Trace-Based Energy Model

To emulate realistic energy-harvesting behavior, we adopt a solar trace-based energy model using publicly available solar irradiance traces from the National Renewable Energy Laboratory (NREL) NSRDB dataset [25]. Each communication round corresponds to one hour, which allows direct mapping between time and harvested solar energy generation patterns. This setting is particularly suitable for solar-powered EH-AIoT systems where energy availability strongly depends on diurnal cycles and environmental conditions.

The harvested energy $H_{i,t}$ follows realistic daytime and nighttime variations derived from the solar traces, with near-zero values during nighttime and higher energy generation during daytime periods. The average harvested energy is approximately 2.4 Wh per round, with peak values reaching 4–5 Wh depending on the trace condition. This configuration reflects a practical EH-AIoT scenario in which the long-term average harvested energy is sufficient for sustainable operation, while temporary low-energy intervals still create significant stability challenges for continual federated learning.

#### 4.1.2. Energy and System Parameters

Although the evaluation is simulation-based rather than prototype-based, the energy parameters are chosen to emulate a small-scale solar-powered edge–device profile.

The energy-related parameters are summarized in Table 2. The battery capacity $C_{\max}$ is set to 240 Wh, which corresponds to a typical small-scale solar-powered edge device. The initial energy is set to 60% of the capacity to avoid bias toward either extreme conditions. The safety threshold $E_{safe}$ is chosen as 25% of the battery capacity, below which the risk of blackout significantly increases.

The target energy level $E^{*}$ is set to 50% of the capacity. This choice is motivated by the need to maintain sufficient buffer against both depletion and overflow, ensuring stable operation under fluctuating energy input.

The training energy coefficient *a* and rehearsal factor ρ are chosen to reflect realistic computation costs observed in edge devices. In particular, ρ=0.8 models the additional overhead caused by mixing replay samples during training. The communication parameters are selected based on typical wireless transmission energy costs.

Overall, the parameter set is designed to satisfy the *energy-neutral condition*, where the long-term average energy consumption does not exceed the harvested energy.

#### 4.1.3. Learning Configuration

We evaluate continual learning performance using CIFAR-10, which is partitioned into two tasks:

- Task 1: Classes 0–4.
- Task 2: Classes 5–9.

The task switch occurs at round 360 out of a total of 720 rounds. This setup enables evaluation of both forward learning (adaptation to new data) and backward transfer (retention of old knowledge).

A lightweight CNN model is used to reflect realistic deployment constraints. The optimizer is SGD with momentum 0.9, and the learning rate is set to 0.01. The batch size is 32. Data is distributed in a non-IID manner, with each client assigned a subset of classes to emulate heterogeneous sensing environments.

We note that this simplified two-task setting is intentionally designed to isolate the effect of energy-aware control on continual learning behavior. More complex multi-task scenarios may introduce additional factors, but the chosen setup allows clear evaluation of the trade-off between energy stability and knowledge retention.

The simulation environment consists of 10 EH-AIoT clients, among which 5 clients participate in each communication round. The total number of communication rounds is set to 720, and the task transition occurs at round 360. This configuration enables evaluation of long-term energy stability and continual learning behavior under realistic time-varying EH conditions.

### 4.2. Baselines

We compare the proposed method with three representative baselines:

- **Fixed**: Constant training intensity and rehearsal ratio. This represents a performance-oriented approach without energy awareness.
- **Threshold-only**: Binary participation based on energy threshold. This represents a stability-oriented approach.
- **Energy-only adaptive**: Adjusts training intensity but keeps rehearsal fixed. This isolates the effect of energy adaptation without continual learning control.

These baselines are designed to represent the major methodological categories discussed in the related work. The Fixed baseline reflects conventional FL or FCL settings with fixed local training and rehearsal configurations. The Threshold-only baseline represents coarse energy-aware participation control used in energy-constrained FL systems. The Energy-only adaptive baseline captures energy-aware training-intensity adaptation without explicit rehearsal control. Therefore, the comparison isolates the contribution of joint control over training intensity and rehearsal under energy-harvesting constraints.

### 4.3. Evaluation Metrics

We evaluate the performance using the following metrics:

- **Blackout Rate (BOR)**: Fraction of rounds where energy depletion occurs.
- **Average Accuracy**: Overall classification accuracy across tasks.
- **Forgetting**: Performance degradation on previous tasks after learning new tasks.
- **Participation Rate**: Fraction of rounds with successful client updates.

These metrics collectively capture system stability, learning performance, and continual learning capability.

### 4.4. Performance Comparison

Table 3 summarizes the overall performance of all methods using quantitative metrics. The proposed method achieves the highest accuracy and the lowest forgetting, while also reducing the blackout rate from 15.4% in the Fixed method and 3.8% in the Energy-only adaptive method to 1.6%. In addition, it achieves the highest participation rate of 84.5%, indicating that the proposed joint control policy improves both learning performance and update continuity.

**Figure 2:**
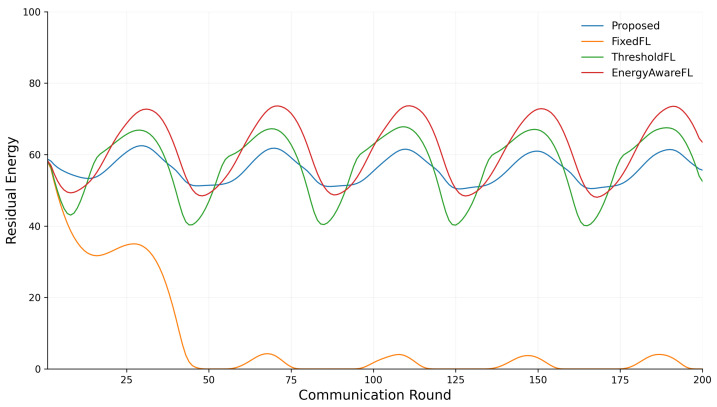
Residual energy dynamics of different methods over communication rounds.

The Fixed method exhibits the lowest overall performance. Although it initially shows rapid improvement, its energy consumption remains high regardless of energy conditions. As shown in Figure 2, this leads to rapid energy depletion and frequent blackout events. These disruptions negatively affect both accuracy and knowledge retention over time.

The Threshold-only method improves energy stability by disabling training when the energy level falls below a predefined threshold. While this prevents complete energy collapse, it introduces frequent interruptions in learning. As a result, the model adapts more slowly and tends to exhibit relatively higher forgetting.

The Energy-only adaptive method alleviates some of these issues by adjusting the training intensity based on the energy state. However, since the rehearsal ratio remains fixed, its ability to retain previously learned knowledge is limited. This results in moderate performance in both accuracy and forgetting.

In contrast, the proposed method jointly controls both training intensity and rehearsal ratio. This enables adaptive balancing between energy consumption and knowledge retention. Consequently, it consistently achieves better quantitative performance across all evaluation metrics, as shown in Table 3. The reported results are averaged over multiple simulation runs, and minor variations were observed across different runs.

### 4.5. Ablation Study

To further examine the contribution of each design component, we conducted an ablation study by selectively removing or modifying key components of the proposed framework. We evaluated three ablated variants: *w/o Rehearsal Control*, in which the rehearsal ratio is fixed and not adaptively adjusted according to the energy state; *w/o Training-Intensity Control*, in which the local training intensity is fixed while rehearsal control is retained; and *w/o Mode-Based Policy*, in which the discrete Risk/Normal/Slack policy is replaced by a continuous energy-dependent adjustment rule. These variants are compared with the full proposed framework.

Table 4 shows the ablation results. Removing rehearsal control increases forgetting because the client can no longer adapt replay usage according to the energy state. Removing training-intensity control increases blackout and reduces participation because the client cannot sufficiently reduce local computation during low-energy periods. Replacing the mode-based policy with a continuous adjustment rule provides reasonable learning performance, but it leads to higher blackout and lower participation due to less robust behavior under fluctuating harvested energy. In contrast, the full proposed framework achieves the best overall balance among accuracy, forgetting, blackout rate, and participation rate.

### 4.6. Energy Stability Analysis

Figure 2 illustrates the residual energy trajectories of different methods over communication rounds under the baseline solar trace-based energy-harvesting setting.

The curves exhibit a clear periodic pattern with an approximately 24-round cycle, reflecting the diurnal nature of solar energy harvesting.

In the proposed method, the residual energy trajectory in Figure 2 is linked to the adaptive learning effort selected by the three operating modes. When the available energy margin is low, the client uses Risk mode with minimum training intensity and no rehearsal. Under moderate energy conditions, Normal mode applies balanced training and rehearsal. When sufficient energy is available, Slack mode increases both training intensity and rehearsal usage. This explains how the proposed method adjusts learning effort according to the available battery state.

The Fixed method shows a rapid decline in energy due to its constant high energy consumption, regardless of the available harvested energy. As a result, the residual energy quickly collapses to the blackout region and remains near zero, indicating unstable and unsustainable operation.

The Threshold-only method maintains the energy above a certain level by disabling training when the residual energy falls below a predefined threshold. This results in a characteristic piecewise trajectory with alternating slopes, where the energy decreases rapidly during active training periods and recovers when training is suspended. Although this mechanism prevents complete depletion, it leads to inefficient energy utilization and frequent interruptions in the learning process.

The Energy-only adaptive method adjusts the training intensity according to the energy state, resulting in smoother energy variations compared to the Threshold-only method. However, since it does not explicitly enforce long-term energy balance, the energy trajectory still exhibits relatively large fluctuations.

In interpreting these energy trajectories, it should be noted that a higher average residual energy does not necessarily indicate better overall performance in continual federated learning. A method may preserve more residual energy by reducing learning activity or limiting participation, but this can degrade model adaptation and knowledge retention. Therefore, the energy trajectory should be interpreted together with the quantitative results in Table 3, including blackout rate, participation rate, accuracy, and forgetting.

In contrast, the proposed method exhibits stable bounded behavior, where the residual energy remains within a controlled range while preserving the underlying periodic pattern. The objective of the proposed method is not to maximize the average residual energy itself, but to avoid energy collapse while maintaining sufficient participation and continual learning capability. This behavior is a direct consequence of the Lyapunov-based control mechanism, which dynamically regulates energy consumption based on the current energy state. As a result, the proposed method achieves energy-neutral operation, prevents long-term energy drift, and provides a better balance between energy stability and learning performance.

### 4.7. Robustness Under Stochastic Energy Variations

To further evaluate the robustness of the proposed framework under practical solar energy fluctuations, we additionally conducted experiments using stochastic harvested-energy variations. Specifically, random perturbations were added to the original solar energy traces in order to emulate temporary environmental fluctuations such as cloud coverage and unstable solar irradiance conditions.

The stochastic harvested energy was modeled as

(18) $$H_{i,t}^{rand}=\max\left(0,H_{i,t}^{base}\left(1+\epsilon_{t}\right)\right),$$

where $H_{i,t}^{base}$ denotes the original solar trace value and $\epsilon_{t}∼N\left(0,0.15^{2}\right)$ represents Gaussian perturbation noise.

Table 5 summarizes the performance under stochastic energy variations. Although all methods experience slight performance degradation due to unstable harvested-energy conditions, the proposed method consistently maintains lower blackout occurrence and better continual learning performance compared to the baseline approaches. These results demonstrate the robustness of the proposed stability-control framework under dynamically changing EH conditions.

### 4.8. Learning Dynamics Analysis

**Figure 3:**
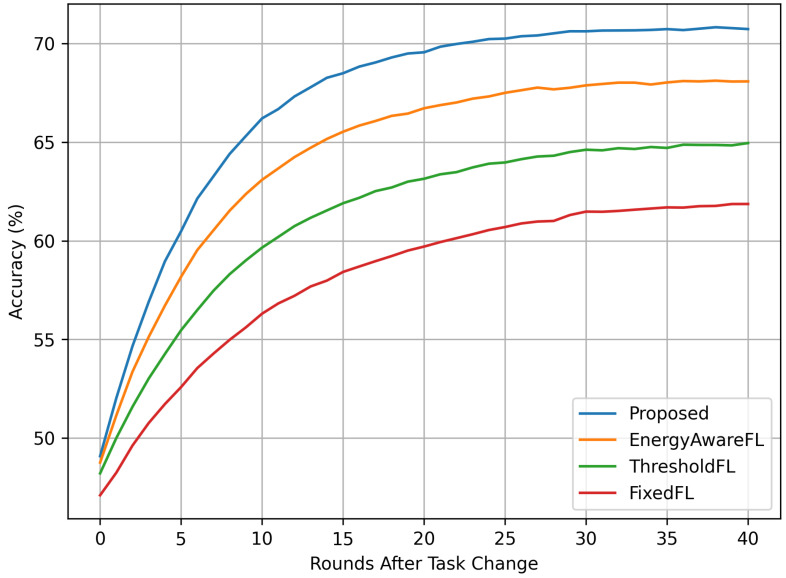
Accuracy evolution after task change.

Figure 3 shows the accuracy evolution after a task change. All methods exhibit a rapid increase in accuracy during the early stages, followed by a gradual saturation.

The Fixed method initially improves quickly but its progress slows down due to unstable training caused by energy depletion. The Threshold-only method shows slower but more stable improvement, reflecting its conservative training behavior.

The Energy-only adaptive method achieves faster adaptation than Threshold-only, but its performance gain is limited by the lack of rehearsal control. In contrast, the proposed method shows both rapid early improvement and stable convergence, resulting in the highest final accuracy.

**Figure 4:**
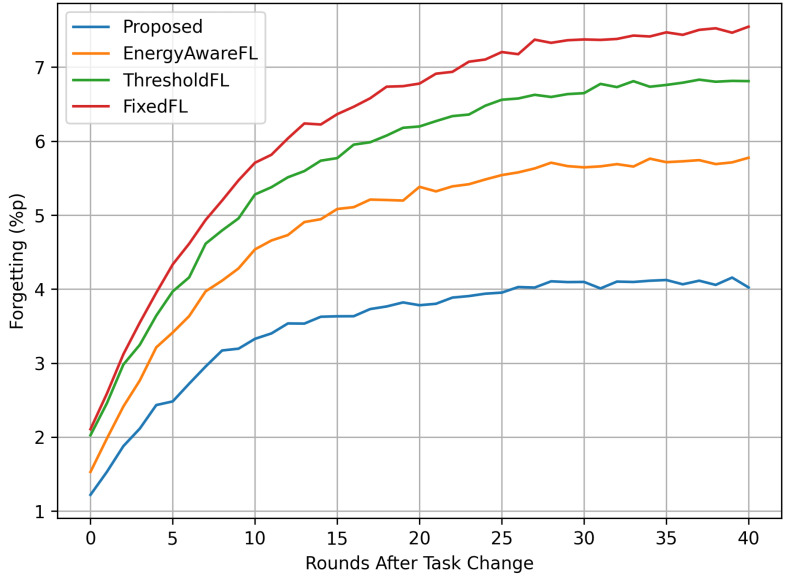
Forgetting behavior after task change.

Figure 4 shows the forgetting behavior after the task change. All methods initially experience an increase in forgetting, which gradually stabilizes.

The Fixed and Threshold-only methods exhibit higher forgetting levels overall, due to unstable or interrupted learning processes. The Energy-only adaptive method reduces forgetting to some extent, but still lacks sufficient control over replay.

The proposed method consistently maintains lower forgetting and stabilizes at a lower plateau. This behavior can be attributed to its adaptive control of rehearsal, which reinforces previously learned knowledge when sufficient energy is available.

Overall, the results indicate that both the timing and intensity of replay are critical for mitigating catastrophic forgetting in energy-harvesting environments.

### 4.9. Reproducibility and Practical Considerations

All parameters used in this study are measurable in real systems. The training energy coefficient can be obtained through micro-benchmarking, and communication energy can be measured through repeated transmissions. Therefore, the proposed framework can be parameterized for real-world EH-AIoT deployments using device-specific measurements. Since the present evaluation is simulation-based, hardware-specific voltage traces, measured power profiles, and photographs of a physical AIoT prototype are not included in this study; these aspects will be addressed in future real-device validation.

The computational overhead of the proposed control policy is lightweight compared with local neural network training. The Fixed baseline does not require adaptive control computation, while the Threshold-only baseline only performs a simple energy-threshold comparison. The Energy-only adaptive baseline additionally adjusts the local training intensity according to the energy state. In comparison, the proposed method performs moving-average harvested-energy estimation, energy-slack computation, two threshold comparisons for mode selection, and direct assignment of the corresponding training intensity and rehearsal ratio. Since the proposed policy does not solve an online optimization problem at each round, the additional control overhead remains negligible relative to the cost of local training and model update transmission.

## 5. Discussion

The experimental results highlight several important insights regarding the design of continual federated learning in energy-harvesting AIoT systems.

First, energy-aware control alone is not sufficient to achieve stable and effective learning. As observed in the Energy-only adaptive method, adjusting the training intensity based on the energy state improves stability compared to fixed training schemes. However, without controlling the rehearsal process, the method cannot effectively mitigate catastrophic forgetting. The ablation study further supports this observation: removing rehearsal control increases forgetting, whereas removing training-intensity control degrades energy stability and participation continuity. These results indicate that learning dynamics, memory retention, and energy-aware control must be considered jointly rather than independently.

Second, simple threshold-based strategies are inadequate for handling time-varying energy conditions. The Threshold-only method maintains system stability by disabling training under low-energy conditions, but this leads to discontinuous learning behavior. As a result, the model experiences delayed adaptation and higher forgetting. This suggests that binary on/off control is too coarse to capture the nuanced trade-offs required in EH environments.

Third, the results demonstrate that the timing of learning is as important as the amount of learning. The proposed method improves performance not merely by increasing computational effort, but by allocating learning and rehearsal operations to periods when energy is available. This adaptive scheduling enables efficient use of harvested energy while preserving knowledge from previous tasks.

Fourth, the energy dynamics analysis confirms that maintaining an energy-neutral operating regime is critical for long-term system sustainability. Methods that ignore energy balance, such as FixedFL, inevitably lead to energy depletion and unstable operation. In contrast, the proposed Lyapunov-based control framework explicitly regulates energy consumption, ensuring that the residual energy remains bounded over time. At the same time, energy stability should not be evaluated only by the average residual energy level. Maintaining a high residual energy level can be achieved by reducing learning activity, but such behavior may lower participation and weaken continual learning performance. Therefore, the proposed method aims to achieve balanced operation by jointly considering bounded energy dynamics, participation continuity, accuracy, and forgetting.

Finally, the proposed three-level control policy provides a practical balance between theoretical optimality and implementation simplicity. While more complex optimization-based controllers could potentially achieve finer control, they are not suitable for resource-constrained edge devices. The mode-based approach achieves comparable behavior with minimal computational overhead, making it more suitable for real-world deployment.

## 6. Conclusions

In this paper, we proposed a stability-controlled continual federated learning framework for energy-harvesting AIoT systems. Unlike conventional approaches that treat energy management and learning optimization separately, the proposed method formulates them as a unified control problem.

To address the inherent trade-off between energy stability and catastrophic forgetting, we introduced a Lyapunov-based control mechanism that jointly regulates local training intensity and rehearsal usage. Furthermore, we designed a lightweight three-level control policy that enables efficient implementation on resource-constrained devices.

Experimental results based on solar energy traces demonstrated that the proposed method significantly improves both energy stability and learning performance. In particular, it maintains bounded energy dynamics while achieving higher accuracy and lower forgetting compared to existing methods.

These results suggest that effective continual learning in EH-AIoT systems requires not only adaptive computation but also energy-aware scheduling of learning processes. The proposed framework provides a practical and principled solution for stable learning in energy-harvesting AIoT systems.

As future work, we plan to extend the proposed framework to more complex scenarios, including heterogeneous devices, stochastic communication environments, and multi-task continual learning settings.

The current study focuses on simulation-based validation using realistic solar energy traces, and practical deployment on real EH-AIoT hardware platforms remains an important direction for future work. Future extensions may also incorporate more advanced solar energy forecasting models using weather information or long-term environmental patterns.

## Figures and Tables

**Table 1:** Technical comparison of related methods and the proposed framework.

Approach	EH	FL	CL/FCL	Rehearsal	Stability Control
Energy-aware FL [13,14]	Partial	Yes	No	No	Partial
EH-FL with scheduling [15]	Yes	Yes	No	No	Partial
Federated continual learning [21,22,23]	No	Yes	Yes	Yes	No
Model-efficiency methods	Partial	Yes	No	No	No
Proposed framework	Yes	Yes	Yes	Yes	Yes

**Table 2:** Simulation Parameters for Energy Harvesting and System Configuration.

Parameter	Description	Value
$C_{\max}$	Battery capacity	240 Wh
$E_{i,0}$	Initial energy	144 Wh
$E_{safe}$	Safety threshold	60 Wh
$E^{*}$	Target energy level	120 Wh
$C_{base}$	Base consumption	0.8 Wh/round
*a*	Training energy per step	0.12 Wh
ρ	Rehearsal overhead factor	0.8
*b*	Communication energy per MB	0.03 Wh
$s_{\Delta }$	Update size	5 MB

**Table 3:** Performance Comparison of Different Methods.

Method	Accuracy (%)	Forgetting (%)	BOR (%)	Participation (%)
Fixed	82.9	7.6	15.4	48.2
Threshold-only	84.6	6.9	5.6	61.7
Energy-only adaptive	86.1	5.8	3.8	72.9
Proposed	87.3	4.1	1.6	84.5

**Table 4:** Ablation study of the proposed framework.

Method	Accuracy (%)	Forgetting (%)	BOR (%)	Participation (%)
w/o Rehearsal Control	86.0	5.9	2.4	81.6
w/o Training-Intensity Control	85.4	4.8	5.1	71.8
w/o Mode-Based Policy	86.5	4.7	2.9	79.5
Proposed	87.3	4.1	1.6	84.5

**Table 5:** Performance under Stochastic Solar Energy Variations.

Method	Accuracy (%)	Forgetting (%)	BOR (%)	Participation (%)
Fixed	78.9	9.8	18.7	42.5
Threshold-only	81.7	8.1	9.4	56.8
Energy-only adaptive	84.0	6.9	5.8	68.3
Proposed	85.4	5.6	3.1	76.4

## Data Availability

The original contributions presented in the study are included in the article, further inquiries can be directed to the corresponding author.
